# Resource Utilization Groups in transitional home care: validating the RUG-III/HC case-mix system in hospital-to-home care programs

**DOI:** 10.1186/s12913-023-10150-1

**Published:** 2023-11-30

**Authors:** Clara Bolster-Foucault, Paul Holyoke

**Affiliations:** 1 Department of Epidemiology, Biostatistics, and Occupational Health, School of Population and Global Health, McGill University, 2001 McGill College, Montreal, QC Canada; 2 SE Research Centre, SE Health, 90 Allstate Parkway, Suite 800, Markham, ON Canada

**Keywords:** Transitional care, Home care, Post-acute care, RUG-III/HC, Resource utilization, Case-mix, interRAI

## Abstract

**Background:**

Transitional hospital-to-home care programs support safe and timely transition from acute care settings back into the community. Case-mix systems that classify transitional care clients into groups based on their resource utilization can assist with care planning, calculating reimbursement rates in bundled care funding models, and predicting health human resource needs. This study evaluated the fit and relevance of the Resource Utilization Groups version III for Home Care (RUG-III/HC) case-mix classification system in transitional care programs in Ontario, Canada.

**Methods:**

We conducted a retrospective analysis of clinical assessment data and administrative billing records from a cohort of clients (*n* = 1,680 care episodes) in transitional home care programs in Ontario. We classified care episodes into established RUG-III/HC groups based on clients’ clinical and functional characteristics and calculated four case-mix indices to describe care relative resource utilization in the study sample. Using these indices in linear regression models, we evaluated the degree to which the RUG-III/HC system can be used to predict care resource utilization.

**Results:**

A majority of transitional home care clients are classified as being Clinically complex (41.6%) and having Reduced physical functions (37.8%). The RUG-III/HC groups that account for the largest share of clients are those with the lowest hierarchical ranking, indicating low Activities of Daily Living limitations but a range of Instrumental Activities of Daily Living limitations. There is notable heterogeneity in the distribution of clients in RUG-III/HC groups across transitional care programs. The case-mix indices reflect decreasing hierarchical resource use within but not across RUG-III/HC categories. The RUG-III/HC predicts 23.34% of the variance in resource utilization of combined paid and unpaid care time.

**Conclusions:**

The distribution of clients across RUG-III/HC groups in transitional home care programs is remarkably different from clients in long-stay home care settings. Transitional care programs have a higher proportion of Clinically complex clients and a lower proportion of clients with Reduced physical function. This study contributes to the development of a case-mix system for clients in transitional home care programs which can be used by care managers to inform planning, costing, and resource allocation in these programs.

**Supplementary Information:**

The online version contains supplementary material available at 10.1186/s12913-023-10150-1.

## Background

Transitional hospital-to-home care programs have emerged as a promising approach to improve care trajectories for older adults following hospitalization in Canada [1]. These programs are designed to provide comprehensive post-acute care to facilitate a safe and timely transition back into the community [2]. They offer a range of medical care and supportive services including nursing care, rehabilitation therapy, social work, and personal support in order to promote recovery and functional independence [3]. Transitional care differs from the existing home health care landscape in Canada in its comparatively narrow focus, defined time-frame, and wide scope of services. A majority of home care in the Canadian context is delivered in long-stay home care, which is designed to support clients with ongoing care needs to remain in the community by providing medical and supportive services over an extended period of time (60 days or longer). In contrast, transitional care is intended reduce delayed hospital discharges by delivering a relatively high intensity of care at home over a defined period (typically 1 to 3 months), with the possibility of transitioning to long-stay home care to receive ongoing care at a lower intensity as needed [4, 5]. Conversely, while short-stay home care has a similar focus and time-frame as transitional care - offering post-acute care for a short period (up to 60 days) - it includes a more limited range of services that focus exclusively on a wound or injury.

A key feature of transitional care programs is the integration of care across a broad spectrum of services, which fosters coordination and continuity of care [6]. Transitional care is effective at reducing the length of hospitalizations, decreasing readmissions and emergency department use following discharge, and reducing the overall cost of care by delivering it in a community setting [7, 8]. They have also been shown to enhance client experiences by increasing quality of life and satisfaction with care [8, 9]. Transitional care aligns with a growing recognition that clients with complex care needs can be cared for at home [10].

The complexity and variability of client needs in transitional care programs requires a robust reimbursement model to enable appropriate care delivery and resource allocation [11]. Long-stay home care in Ontario, Canada, is typically funded through a fee-for-service (FFS) payment model in which healthcare provider reimbursements are based on fees for individual services [12]. FFS models incentivise care volume and are susceptible to care fragmentation as providers are reimbursed separately for their services, which may lead to over-provision of care and poor care coordination [13, 14]. In response to this, there is a growing interest to implement bundled care pricing models in home care and post-acute care [15]. In bundled care models, healthcare providers are compensated a fixed rate for each extended episode of care, regardless of each client’s individual level of clinical complexity and resource utilization [16]. These models contribute to value-based care by aligning economic incentives with efficiency, responsiveness, and care coordination [17]. However, the success of programs that use these funding models depends on the prospective calculation of appropriate reimbursement rates.

Case-mix classification systems aim to predict resource needs and utilization within clinical populations by classifying home care clients into defined groups based on key predictive characteristics. Ideal case-mix systems are grounded in clinically meaningful descriptions of clients in a specific care setting and reflect resource utilization over a relevant time period (e.g. per diem, weekly, throughout a defined clinical episode, etc.) [18]. These schemas can assist with evaluations of the quality and cost of care, inform optimal resource allocation, and facilitate evaluations and comparisons across clients, programs, and institutions [19]. Case-mix indices (CMIs) that reflect relative resource utilization across groups can be derived from these classification systems and can be used to structure funding models in healthcare [18]. Case-mix based funding models facilitate responsive care delivery and ensure that providers are reimbursed appropriately [20]. Accurate and efficient case-mix systems are important tools for calculating prospective payments and have been used to support bundled care models in home care and post-acute care settings [21].

**Fig. 1:**
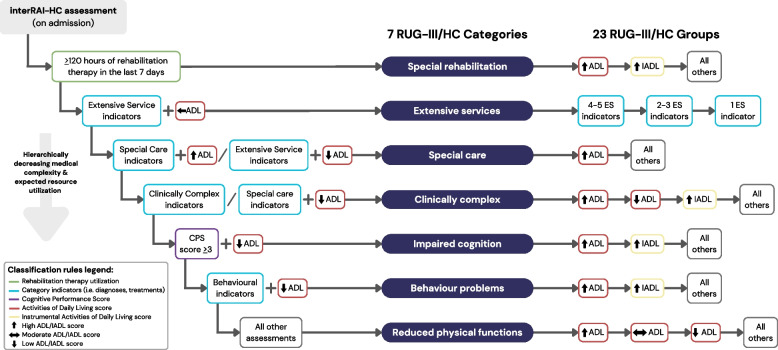
RUG-III/HC groups and classification hierarchy

The Resource Utilization Groups version III for Home Care (RUG-III/HC) case-mix classification system is widely used in home care settings in Canada [22, 23]. This system was originally developed for use in long-stay home care in the United States by Björkgren et al. in 2000 [22] and was further refined and validated in the Canadian context by Poss et al. in 2008 [23]. The RUG-III/HC classifies home care clients into 7 categories based on their clinical characteristics and resource utilization and further subdivides them into 23 groups according to their level of functional dependency as measured by Activities of Daily Living (ADL) and Instrumental Activities of Daily Living (IADL) (Fig. 1) [22]. The groups are hierarchically ranked to reflect decreasing clinical complexity and expected resource utilization.

The RUG-III/HC leverages routinely collected data from client assessments which facilitates its implementation and relevance for care planning and evaluation. The classification algorithm was originally designed using data from the Resident Assessment Instrument – Home Care (RAI-HC), a standardized client assessment instrument completed by clinicians to inform home care planning and delivery. An updated assessment instrument, the interRAI Home Care (interRAI-HC), was published in 2012 and has been widely implemented in long-stay home care settings in Canada [24]. The interRAI-HC includes information on each client’s health status, diagnoses, treatments, cognition, functional status, and social context. These assessments are highly reliable within clients over time and across assessors [25]. The RAI-HC and interRAI-HC were developed by InterRAI – an international consortium of researchers who have published comprehensive assessment instruments for clients with complex care needs or in specific care settings [26].

The RUG-III/HC case-mix classification system has significant potential to inform care planning and resource allocation in transitional hospital-to-home care programs. However, clients receiving care in transitional home care programs may be substantially different from those in long-stay home care. The landscape of home care in Canada has also evolved significantly since the RUG-III/HC was initially validated [27, 28]. Furthermore, the RUG-III/HC has not yet been validated using the updated interRAI-HC assessment instrument [29]. Given the potential differences in client population and care processes between transitional home care and long-stay home care, changes in the home care context, and the utilization of an updated client assessment tool, there is a need to examine the relevance of the RUG-III/HC in transitional home care programs.

## Methods

### Study aim

Our objective was to examine the applicability of the existing RUG-III/HC case-mix classification system to clients in transitional hospital-to-home care programs in Canada. We evaluated the distribution of clients across RUG-III/HC groups in transitional care programs and examined the reliability of the RUG-III/HC case-mix groups for predicting care resource utilization.

### Study design, setting & population

We conducted a retrospective analysis of a cohort of clients from a convenience sample of eight 10–16-week transitional hospital-to-home care programs operated by one home care provider organization in the province of Ontario. These programs were selected for inclusion in consultation with an advisory group of program managers. They represent a large proportion of the transitional home care programs currently in operation in the province and are all relatively homogeneous in their scope, referral patterns, intended length, and time-frame. The full study sample includes data from 1,680 completed care episodes with complete assessments from 2019 to 2022.

### Sample selection

Given the diverse population of clients in transitional home care programs, principles of person-centered care often require deviations from an idealized care pathway to respond to individual needs and circumstances. As such, the full study sample includes some clients with unusually short or long care episodes. The inclusion of these atypical cases in the analysis has significant implications for the interpretation of the RUG-III/HC case-mix groupings [30]. At the request of our advisory group of program managers, we created a second study sample that is more representative of the intended length of transitional programs in order to align with their operational needs. To do so, we excluded outliers with the lowest 1% and highest 5% length of stay in their care episode, resulting in an operational sample of 1,575 care episodes (a 6.37% reduction from the full sample).

These two samples facilitate complementary utilizations of the RUG-III/HC case-mix system. The full sample reflects actual care utilization patterns in transitional care programs, which is most useful for individual care planning and cross-program comparisons. In contrast, the operational sample reflects care utilization patterns of clients whose care episodes are more closely aligned with the intended scope and length of the transitional care programs, which facilitates program planning and calculating prospective payment rates.

### Data sources

#### Clinical data

Information on each client’s clinical characteristics at the beginning of their care episode was obtained from interRAI-HC assessments completed upon admission to the transitional care program.

#### Resource utilization data

Information on paid care resource utilization throughout each care episode was drawn from administrative billing records. These data define the start and end date of the care episode and include the total number of hours that each client received care services from a registered practical nurse (RPN), registered nurse (RN), occupational therapist (OT), physical therapist (PT), speech-language pathologist (SLP), social worker (SW), registered dietician (RD), physical therapy assistant (PTA), and personal support worker (PSW) throughout their care episode. Complete billing records were available for 1,251 care episodes in the full sample and 1,184 care episodes in the operational sample.

Information on unpaid caregiving throughout each care episode was drawn from interRAI-HC assessment data, in which clients are asked to self-report the number of hours of unpaid care they received in the preceding 3 days. This data was retrieved from assessments completed at admission as well as discharge.

### Statistical analysis

#### RUG-III/HC classification

We first classified each care episode into one of the existing 23 RUG-III/HC groups using the established hierarchical classification algorithm developed for long-stay home care settings [23]. We then examined the distribution of care episodes across the 23 RUG-III/HC groups in the full and operational samples and within each of the eight transitional care programs.

#### Case-mix indices

To describe trends in resource utilization of clients admitted to transitional care programs, we derived four case-mix indices (CMIs) which represent the mean paid care time and cost as well as combined paid and unpaid care time and cost for clients in each RUG-III/HC group relative to the overall population. Although the RUG-III/HC is primarily used to inform care planning and resource allocation in paid care settings, the availability of unpaid caregiving has significant implications for home care clients’ health and quality of life which can, in turn, influence their utilization of paid care resources [31]. The inclusion of unpaid care was an important innovation in the original derivation of the RUG-III/HC which has been repeatedly shown to improve its ability to predict resource utilization long-stay home care [22, 23]. This additional context supports a more complete understanding of clients’ care needs.

The number of hours of paid care during each care episode was calculating by aggregating the hours billed by each type of healthcare professional from billing records. To calculate the overall cost of paid care throughout a care episode, hourly billing rates were obtained for each type of healthcare professional. These represent the fair market value of their labour including their hourly wage and indirect costs relating to their employment (e.g., benefits, administrative costs). We standardized these billing rates by the hourly rate of a PSW to create a set of standardized cost weights. PSWs were selected as the reference group for this calculation as they provide the largest proportion of paid care to clients in home care settings [32]. We calculated the total cost of paid care during each care episode by multiplying the number of hours of care that were delivered by each type of healthcare provider by their respective standardized cost weight and aggregating the weighted cost.

We lacked data on the exact number of hours of unpaid caregiving that each client received throughout their care episode. Total hours of unpaid caregiving were therefore estimated by calculating the mean hours of unpaid care reported by clients on interRAI-HC assessments at admission and at discharge and multiplying by the length of the care episode. The cost of unpaid caregiving must also be estimated based on assumptions about the economic value of unpaid care [33]. Using a replacement cost valuation approach, the cost of unpaid care can be estimated by substituting the market rate of a paid healthcare provider who would be required to deliver the same level of care in the absence of an unpaid caregiver [34]. In Ontario home care settings, the care provided by PSWs, unregulated health care providers who provide care under the supervision of a nurse, is aligned with the care provided by unpaid caregivers and is therefore an appropriate choice for costing a replacement worker [32, 35]. We elected to assign the full cost of a PSW (i.e. a standardized weight of 1.00) to each hour of unpaid care in order to acknowledge the value of unpaid caregiving in home care contexts.

Using these data, we calculated the mean and standard deviation of four resource utilization measures within each RUG-III/HC group: 1) paid care time, 2) paid care cost, 3) paid and unpaid care time, 4) paid and unpaid care cost. Four corresponding CMIs were derived by dividing each group’s mean by the overall population mean, representing relative resource utilization across RUG-III/HC groups.

#### Coefficient of variation

We examined within-group homogeneity of care resource utilization by calculating the coefficient of variation (CV) associated with the four CMIs in each RUG-III/HC group and in the overall study population.

#### Variance explained

To evaluate the relevance of the RUG-III/HC groups for predicting resource utilization in transitional care programs, we modelled each client’s care resource utilization as a function of the four CMIs using linear regression and extracted the variance explained (*R*^2^) of each model [21].

## Results

The demographic and clinical characteristics of clients in the full study sample are described in Table 1. Clients’ mean age was 76.36 (median: 79, range: 17–110). A majority were female (59.25%), were married or had a partner (41.85%), and lived alone (38.33%). The most common diagnoses among clients in the sample were diabetes mellitus (30.02%), coronary heart disease (24.23%), and cancer (19.73%). Care episodes had a mean length of 99.49 days (median: 112, range: 3–622) in the full sample and 96.83 days (median: 111, range: 12–141) in the operational sample. Clients received a mean of 74.05 hours of paid care and 350.36 hours of unpaid care during their care episode, with a majority of paid caregiving being delivered by PSWs, RPNs and RNs.

**Table 1:** Full sample population characteristics

**Client characteristics**	**N (%) / Mean (SD)**
Care episodes	1804 (-)
Unique clients	1787 (-)
Age	76.35 (12.94)
Sex	
Female	1063 (59.25%)
Male	730 (40.69%)
Marital status	
Married/Partner	750 (41.85%)
Divorced/Separated	257 (14.34%)
Widowed	571 (31.86%)
Never married	214 (11.94%)
Living arrangement	
Lives alone	685 (38.33%)
Lives with spouse/partner only	505 (28.26%)
Lives with spouse/partner and other(s)	192 (10.74%)
Lives with child(ren)	270 (15.11%)
Lives with other relative(s)	88 (4.92%)
Lives with non-relative(s)	47 (2.63%)
Most common diagnoses	
Diabetes mellitus	508 (30.02%)
Coronary heart disease	410 (24.23%)
Cancer	334 (19.73%)
Congestive heart failure	294 (17.38%)
Other fracture (last 30 days)	264 (15.57%)
Depression	262 (15.50%)
Stroke/Cerebrovascular accident	255 (15.07%)
Anxiety	219 (12.96%)
Hypertension	202 (12.09%)
Chronic obstructive pulmonary disease	201 (11.88%)
Days in care episode	99.49 (35.90)
Hours of paid care per episode	74.05 (64.41)
Registered practical nurse (RPN)	13.96 (12.51)
Registered nurse (RN)	12.84 (13.59)
Occupational therapist (OT)	4.63 (5.09)
Physical therapist (PT)	5.50 (5.35)
Speech language pathologist (SLP)	0.97 (3.21)
Social worker (SW)	3.59 (5.73)
Registered dietitian (RD)	1.93 (2.89)
Physical therapy assistant (PTA)	9.06 (9.60)
Personal support worker (PSW)	41.34 (52.47)
Hours of unpaid care per episode	350.36 (572.66)

The distribution of transitional home care clients across the 7 RUG-III/HC categories and 23 groups in the full and operational study samples is described in Table 2. A large majority of clients were classified into only two of the seven categories: Clinically complex (41.6%) and Reduced physical functions (37.8%). A moderate proportion of clients were classified in Rehabilitation (10.4%) and Special care (5.8%), and relatively few were classified in the Behaviour problems (2.1%), Impaired cognition (2.0%), or Extensive services (0.3%) categories. Notably, the results of the full sample and operational sample are nearly identical, indicating that outliers in care episode duration were not disproportionately distributed among RUG-III/HC groups compared to clients with more typical care episode durations.

**Table 2:** Classification of transitional care clients in RUG-III/HC groups

**RUG-III/HC Group**	Full sample	Operational sample
	% (n)	% (n)
**Rehabilitation**	**10.4 (174)**	**10.4 (164)**
RB	1.0 (17)	1.1 (17)
RA2	2.0 (34)	2.2 (34)
RA1	7.3 (123)	7.2 (113)
**Extensive services**	**0.3 (5)**	**0.3 (4)**
SE3	0.0 (0)	0.0 (0)
SE2	0.3 (5)	0.3 (4)
SE1	0.0 (0)	0.0 (0)
**Special care**	**5.8 (97)**	**5.8 (91)**
SSB	0.3 (5)	0.3 (4)
SSA	5.5 (92)	5.5 (87)
**Clinically complex**	**41.6 (699)**	**41.3 (651)**
CC	2.4 (41)	2.3 (37)
CB	5.3 (89)	5.4 (85)
CA2	20.1 (338)	20.1 (317)
CA1	13.8 (231)	13.5 (212)
**Impaired cognition**	**2.0 (34)**	**2.1 (33)**
IB	0.6 (10)	0.6 (9)
IA2	1.4 (24)	1.5 (24)
IA1	0.0 (0)	0.0 (0)
**Behaviour problems**	**2.1 (36)**	**2.1 (33)**
BB	0.4 (6)	0.4 (6)
BA2	1.1 (19)	1.1 (17)
BA1	0.7 (11)	0.6 (10)
**Reduced physical functions**	**37.8 (635)**	**38.0 (599)**
PD	2.7 (46)	2.7 (43)
PC	1.3 (21)	1.3 (20)
PB	3.5 (59)	3.7 (59)
PA2	16.1 (270)	16.3 (256)
PA1	14.2 (239)	14.0 (221)
**Total**	**100.00 (1680)**	**100.00 (1575)**

Within the RUG-III/HC categories, the proportion of clients classified in each group ranged from 20.1% in the second lowest Clinically complex group (CA2) to 0.3% in the highest Special care group (SSB) and second highest Extensive services group (SE2) – excluding three groups (SE3, SE1, and IA1) into which no clients were classified. Across almost all RUG-III/HC categories, the groups that account for the largest proportion of clients are those with lower hierarchical ranking, indicating low ADL limitations but a range of IADL limitations. This trend is particularly evident in the two largest RUG-III/HC categories: Clinically complex and Reduced physical functions, in which a majority of clients are classified into groups with low ADL limitations but mild to severe IADL limitations (CA2: 20.1% and PA2: 16.1%) and groups with low ADL limitations and no IADL limitations (CA1: 13.8% and PA1: 14.2%). Estimates for groups into which fewer than 10 clients have been classified are likely unstable and should be interpreted with caution.

**Fig. 2:**
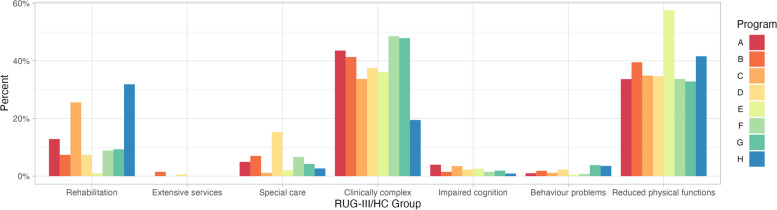
Distribution of clients in RUG-III/HC groups across transitional programs

There were notable differences in the distribution of clients in RUG-III/HC groups across transitional home care programs in the study sample (Fig. 2). There are outliers in some programs with respect to the proportion of Rehabilitation and Reduced physical functions clients, as well as heterogeneity in the proportion of Special care and Clinically complex clients. This may reflect true differences in the client populations, differences in referral patterns between acute care settings referring to each of these programs, or differences in the use of and availability of health human resources across programs.

The four CMIs for each of the RUG-III/HC groups in the operational sample are presented in Table 3. The CMIs are measures of relative resource utilization and are expected to reflect the hierarchical ordering of the RUG-III/HC through a pattern of decreasing magnitude across the categories and groups. Within each of the seven overarching RUG-III/HC categories, the CMIs for paid care and combined paid and unpaid care perform largely as expected, showing a decreasing pattern of resource utilization across groups for both care time and cost. For example, within the Clinically complex category the CMI for paid care time displays a clear decreasing pattern ranging from 1.68 in the highest hierarchical group (CC) to 0.81 in the lowest hierarchical group (CA1). However, the expected decreasing pattern is not readily apparent across the seven RUG-III/HC categories, particularly when accounting for paid care only. This is evidenced by the fact that the range of CMIs within the seven RUG-III/HC categories shows a significant degree of overlap.

**Table 3:** Case-mix indices (CMI) and coefficients of variation (CV) of operational sample

**RUG-III/HC Group**	**n**	**Paid Care Time** **CMI (CV)**	**Paid Care Cost** **CMI (CV)**	**Paid + Unpaid Care Time** **CMI (CV)**	**Paid + Unpaid Care Cost** **CMI (CV)**
**Overall**	1184	1.00 (0.87)	1.00 (0.86)	1.00 (1.34)	1.00 (1.32)
**Rehabilitation**					
RB	15	1.14 (0.91)	1.19 (0.91)	3.06 (0.69)	3.04 (0.68)
RA2	26	1.03 (0.62)	0.94 (0.59)	2.31 (1.07)	2.27 (1.07)
RA1	85	1.21 (0.86)	1.18 (0.87)	1.05 (1.25)	1.04 (1.24)
**Extensive services**					
SE3	0	NA	NA	NA	NA
SE2	4	0.88 (0.43)	0.92 (0.34)	1.27 (1.19)	1.27 (1.16)
SE1	0	NA	NA	NA	NA
**Special care**					
SSB	4	1.83 (0.52)	1.71 (0.52)	3.00 (0.69)	2.95 (0.67)
SSA	70	1.16 (0.79)	1.20 (0.77)	1.03 (1.20)	1.04 (1.18)
**Clinically complex**					
CC	32	1.68 (0.78)	1.70 (0.78)	1.68 (1.12)	1.69 (1.11)
CB	61	1.32 (0.74)	1.26 (0.76)	1.65 (1.13)	1.63 (1.13)
CA2	233	0.99 (0.82)	1.00 (0.81)	1.00 (1.34)	1.00 (1.32)
CA1	161	0.81 (0.75)	0.80 (0.76)	0.51 (1.15)	0.51 (1.12)
**Impaired cognition**					
IB	8	1.06 (0.85)	1.02 (0.72)	2.91 (0.82)	2.87 (0.81)
IA2	18	1.41 (0.81)	1.35 (0.75)	1.36 (1.17)	1.34 (1.16)
IA1	0	NA	NA	NA	NA
**Behaviour problems**					
BB	3	2.07 (0.28)	2.17 (0.28)	0.86 (0.61)	0.89 (0.60)
BA2	13	0.68 (0.54)	0.73 (0.56)	1.41 (1.18)	1.40 (1.16)
BA1	6	0.92 (0.59)	0.96 (0.58)	0.54 (0.54)	0.55 (0.53)
**Reduced physical functions**					
PD	35	1.39 (0.96)	1.40 (0.90)	1.15 (1.17)	1.16 (1.16)
PC	18	0.88 (0.80)	0.86 (0.73)	0.95 (0.90)	0.95 (0.88)
PB	40	1.33 (0.71)	1.34 (0.67)	1.27 (1.04)	1.27 (1.03)
PA2	188	0.87 (0.82)	0.86 (0.80)	0.83 (1.39)	0.82 (1.37)
PA1	164	0.76 (0.88)	0.76 (0.90)	0.52 (1.10)	0.52 (1.08)

The four CMIs and their corresponding CVs in the full sample are nearly identical (Additional file 1). However, the decreasing hierarchical pattern across RUG-III/HC groups is less apparent and the CVs for some groups are slightly larger, indicating marginally lower within-group homogeneity. As above, estimates based on fewer than 10 observations are unstable and should be interpreted with caution.

The variance in care resource utilization that is explained by the RUG-III/HC case-mix classifications (*R*^2^) is summarized in Table 4 using two modelling strategies: CMI alone and CMI with a program indicator variable. Across the four measures of care resource utilization, variance explained is similar for care time and cost. However, the variance explained is markedly higher when accounting for both paid and unpaid care, highlighting the importance of unpaid caregiving in post-acute home care contexts [36]. Adding the program indicator variable to the model also increased the variance explained, reflecting the heterogeneity in client populations across programs noted in Fig. 2. Finally, the variance in care resource utilization that is explained by the RUG-III/HC groups is higher in the operational sample as opposed to the full sample, which confirms that clients with unusually short or long care episodes are outliers in terms of their care resource utilization.

**Table 4:** Variance in care resource utilization explained by RUG-III/HC groups

**Resource Utilization Measure**	**CMI only**		**CMI + Program Indicator**	
	Full sample	Operational sample	Full sample	Operational sample
Paid care time	6.89%	7.53%	9.77%	10.10%
Paid care cost	7.04%	7.78%	9.47%	9.64%
Paid + Unpaid care time	13.26%	13.53%	21.88%	23.34%
Paid + Unpaid care cost	13.28%	13.56%	21.79%	23.21%

## Discussion

Transitional hospital-to-home home care programs are designed to support clients following a hospital discharge by promoting the recovery or maintenance of functional independence. This analysis examined the applicability of the RUG-III/HC case-mix system, which was developed for long-stay home care settings, to clients in transitional home care. The results suggest that clients in transitional care programs differ from those in traditional long-stay home care. A majority of clients in transitional care were classified in the Clinically complex (41.6%) and Reduced physical functions (37.8%) RUG-III/HC groups. Although these same two groups represent the largest share of clients in long-stay home care as reported by Poss et al. in their validation of the RUG-III/HC in the Canadian home care context in 2008, their relative prevalence is reversed as more than half of long-stay home care clients were classified as having Reduced physical functions (56.1%) and the proportion of clients classified as Clinically complex was less than half that in transitional care programs (21.2%) [23]. This finding reflects the fact that clients in transitional care programs are referred by hospitals for post-acute care rather than being referred through traditional home care channels. This population is therefore significantly more likely to have experienced an acute health event which suggests the requirement for more complex medical care at home. However, these results may also reflect bias in referral patterns from hospitals.

Another notable difference is that transitional home care programs have fewer clients with Impaired cognition (2.0%) compared to long-stay home care (12.8%) [23]. This may reflect a growing tendency for clients with cognitive impairments to be referred to institutional long-term care facilities or indicate that clients with impaired cognition in transitional care programs have concurrent clinical needs that result in them being classified into higher hierarchical RUG-III/HC groups. Conversely, transitional care programs have a higher proportion of clients in Rehabilitation (10.4%) compared to long-stay home care (6.8%) [23]. This may reflect the fact that these programs are contractually obliged to deliver rehabilitation services to all clients regardless of need, resulting in a higher likelihood that a client will meet the criteria for classification into this group.

Within each of the RUG-III/HC categories, transitional home care clients were more likely to be classified into the two lowest hierarchical groups indicating low ADL limitations but a range of IADL limitations. In contrast, long-stay home care clients were more likely to be classified into the lowest hierarchical group which indicates both low ADL and IADL limitations. Given their post-acute status, clients in transitional care may experience a wider range of IADL limitations. Functional status has been found to be a significant driver of variance explained in case-mix models in long-term care settings, which may influence the applicability of the RUG-III/HC in transitional care [19].

The predictive power of case-mix systems in home care settings is highly variable and reported variance explained in other studies has ranged from 14% in to 54.3% [37]. The RUG-III/HC case-mix system predicted a moderate amount of variance in care resource utilization in transitional care clients, explaining 23.34% of the variance in paid and unpaid care time in the operational sample and 21.88% in the full sample. This is considerably lower than the 37.3% variance explained reported by Poss et al. in 2008 [23]. However, Poss et al. noted that the variance explained was significantly higher for long-standing cases (44.5%) as opposed to newly opened cases (28.4%) [23]. As all care episodes in our study sample are newly opened transitional care cases, the latter group might serve as a more valid comparison. In line with this, a systematic review of case-mix schemas in home care settings found that variance explained increases as the length of care episodes increase [37]. This suggests that a moderate variance explained is expected in time-limited programs such as transitional home care.

Although it was originally developed and validated for long-stay home care settings, the RUG-III/HC case-mix system has the potential to support informed decision-making in transitional home care. By leveraging routinely-collected interRAI-HC assessment data, the RUG-III/HC can be used to guide care and program planning and evaluation. The results from the full and operational study samples can inform complementary uses of the RUG-III/HC. The full sample contains data from all completed care episodes regardless of their length, which renders the results most useful to individual care planning as well as program evaluation. The operational sample reflects an idealized client population, which is most useful for prospective program planning. However, there is an opportunity to refine the RUG-III/HC case-mix algorithm to improve its relevance for predicting care resource utilization in transitional programs. This may be achieved by modifying the classification structure and hierarchy to more accurately reflect the care delivered in transitional programs or by incorporating additional variables in the classification algorithm that may be important for explaining care resource utilization in transitional care, such as specific diagnoses (e.g., Alzheimer’s, stroke, depression) or health events (e.g. falls), measures of mobility and self-management, and social factors that influence unpaid caregiving.

### Limitations

The results of this analysis should be considered in the context of its limitations. First, 124 care episodes were excluded from the study sample due to missing interRAI-HC assessment data and missing billing data for 429 care episodes resulted in their exclusion from the CMI calculation. If this missing data is non-random with respect to their clinical characteristics and resource utilization, the RUG-III/HC distribution and CMI calculations may be biased. Similarly, the possibility of misclassification due to errors or bias in the interRAI-HC assessment data cannot be eliminated, even if it is unlikely [25, 38]. Similarly, as we lacked data on the exact number of hours of unpaid caregiving that each client received during their care episode, we based our analysis on an approximate calculation using admission and discharge data. Comparisons of the mean hours of unpaid caregiving that each client reported at admission and discharge show that the number of hours of unpaid caregiving is relatively stable throughout the entire care episode. However, trajectories of unpaid caregiving following an acute health event can be highly variable, which may result in an over- or under-estimation of unpaid caregiving hours [39].

The contractual structure of transitional care programs as well as the timing of interRAI-HC assessments on admission to a program may also influence the classification of clients into RUG-III/HC groups. The transitional care programs examined in this study were designed to deliver a high degree of rehabilitation services and nursing care which contribute to RUG-III/HC classification and CMI calculation, respectively. In addition, several questions on the interRAI-HC require assessors to base themselves on retrospective answers, including the number of hours of rehabilitation minutes received in the past 7 days, ADL/IADL performance in the past 3 days, and unpaid caregiving in the past 3 days. Transitional care programs require interRAI-HC assessments to be completed soon after hospital discharge and admission to the transition program. However, if admission interRAI-HC assessments are completed too soon after hospital discharge, they may miss some elements of care that are relevant for RUG-III/HC classification.

## Conclusions

As healthcare systems continue to evolve and the Canadian population ages, there is a growing need to provide care for people in their homes and communities. The results of this analysis show that the profile of clients receiving post-acute care in transitional hospital-to-home care programs is remarkably different from our understanding of the profile of clients in long-stay home care settings. Transitional home care clients have a greater degree of medical complexity and a wider range of IADL limitations.

Despite the differences in client population, the RUG-III/HC case-mix classification system has the potential to inform individual care delivery, program planning and evaluation, prospective calculation of bundled care pricing, and strategies to optimally allocate human resources in transitional care programs. With a better understanding of the distribution of care needs in transitional care, these programs will be better equipped to deliver appropriate person-centered care. These findings contribute to the ongoing evaluation of the RUG-III/HC classification system and open the potential opportunity to improve the system for different and newer forms of home care programs. Such classification systems can and should be used to inform the development of evidence-based policies and practices for transitional hospital-to-home care in Canada.

### Supplementary Information

**Additional file 1.** Case-mix indices (CMI) and coefficients of variation (CV) of full sample.

## Data Availability

The datasets analysed during the current study are not publicly available due to institutional policies protecting individual client privacy and confidentiality but are available from the corresponding author on reasonable request.

## Abbreviations

ADL: Activities of daily living
CMI: Case-mix index
CV: Coefficient of variation
FFS: Fee-for-service
IADL: Instrumental activities of daily living
interRAI-HC: InterRAI Home Care
OT: Occupational therapist
PT: Physical therapist
PTA: Physical therapy assistant
PSW: Personal support worker
RAI-HC: Resident Assessment Instrument – Home Care
RD: Registered dietician
RN: Registered nurse
RPN: Registered practical nurse
RUG-III/HC: Resource Utilization Groups version III for Home Care
SLP: Speech-language pathologist
SW: Social worker
