# Supplementary material for: Resource Utilization Groups in transitional home care: validating the RUG-III/HC case-mix system in hospital-to-home care programs
Source: BMC Health Serv Res. 2023 Nov 30;23:1324. doi: 10.1186/s12913-023-10150-1 (PMC10687885; doi:10.1186/s12913-023-10150-1)
Supplement: Supplementary file 1 — Additional file 1. Case-mix indices (CMI) and coefficients of variation (CV) of full sample. [file 12913_2023_10150_MOESM1_ESM.docx]

# ADDITIONAL FILES

## Additional file 1 Case-mix indices (CMI) and coefficients of variation (CV) of full sample

| **RUG-III/HC Group** | **n** | **Paid Care Time** CMI (CV) | **Paid Care Cost** CMI (CV) | **Paid + Unpaid Care Time** CMI (CV) | **Paid + Unpaid Care Cost** CMI (CV) |
| --- | --- | --- | --- | --- | --- |
| **Overall** | 1251 | 1.00 (0.87) | 1.00 (0.86) | 1.00 (1.39) | 1.00 (1.37) |
| **Rehabilitation** |  |  |  |  |  |
| RB | 15 | 1.13 (0.91) | 1.18 (0.91) | 3.03 (0.69) | 3.01 (0.68) |
| RA2 | 26 | 1.03 (0.62) | 0.94 (0.59) | 2.28 (1.07) | 2.24 (1.07) |
| RA1 | 89 | 1.22 (0.84) | 1.19 (0.84) | 1.01 (1.25) | 1.01 (1.23) |
| **Extensive services** |  |  |  |  |  |
| SE3 | 0 | NA | NA | NA | NA |
| SE2 | 5 | 0.73 (0.64) | 0.75 (0.61) | 1.06 (1.30) | 1.05 (1.27) |
| SE1 | 0 | NA | NA | NA | NA |
| **Special care** |  |  |  |  |  |
| SSB | 5 | 1.49 (0.74) | 1.40 (0.74) | 2.43 (0.87) | 2.40 (0.86) |
| SSA | 75 | 1.14 (0.79) | 1.18 (0.77) | 1.03 (1.20) | 1.03 (1.18) |
| **Clinically complex** |  |  |  |  |  |
| CC | 36 | 1.62 (0.83) | 1.65 (0.83) | 1.56 (1.16) | 1.56 (1.15) |
| CB | 62 | 1.34 (0.74) | 1.28 (0.75) | 1.62 (1.12) | 1.61 (1.12) |
| CA2 | 246 | 1.01 (0.81) | 1.02 (0.80) | 1.09 (1.41) | 1.08 (1.39) |
| CA1 | 172 | 0.83 (0.77) | 0.82 (0.79) | 0.50 (1.14) | 0.50 (1.11) |
| **Impaired cognition** |  |  |  |  |  |
| IB | 9 | 1.25 (0.81) | 1.21 (0.74) | 3.63 (0.87) | 3.58 (0.87) |
| IA2 | 18 | 1.40 (0.81) | 1.34 (0.75) | 1.34 (1.17) | 1.33 (1.16) |
| IA1 | 0 | NA | NA | NA | NA |
| **Behaviour problems** |  |  |  |  |  |
| BB | 3 | 2.06 (0.28) | 2.15 (0.28) | 0.85 (0.61) | 0.88 (0.60) |
| BA2 | 13 | 0.67 (0.54) | 0.72 (0.56) | 1.39 (1.18) | 1.39 (1.16) |
| BA1 | 6 | 0.91 (0.59) | 0.95 (0.58) | 0.53 (0.54) | 0.55 (0.53) |
| **Reduced physical functions** |  |  |  |  |  |
| PD | 38 | 1.33 (0.98) | 1.34 (0.92) | 1.28 (1.41) | 1.28 (1.39) |
| PC | 18 | 0.88 (0.80) | 0.85 (0.73) | 0.94 (0.90) | 0.94 (0.88) |
| PB | 40 | 1.32 (0.71) | 1.33 (0.67) | 1.25 (1.04) | 1.25 (1.03) |
| PA2 | 199 | 0.88 (0.81) | 0.87 (0.80) | 0.78 (1.41) | 0.78 (1.39) |
| PA1 | 176 | 0.76 (0.89) | 0.76 (0.91) | 0.51 (1.11) | 0.52 (1.09) |
